# Ultrawide Bandgap and High Sensitivity of a Plasmonic Metal-Insulator-Metal Waveguide Filter with Cavity and Baffles

**DOI:** 10.3390/nano10102030

**Published:** 2020-10-15

**Authors:** Yuan-Fong Chou Chau, Chung-Ting Chou Chao, Hung Ji Huang, Muhammad Raziq Rahimi Kooh, Narayana Thotagamuge Roshan Nilantha Kumara, Chee Ming Lim, Hai-Pang Chiang

**Affiliations:** 1 Centre for Advanced Material and Energy Sciences, Universiti Brunei Darussalam, Tungku Link, Gadong BE1410, Brunei; chernyuan@hotmail.com (M.R.R.K.); roshan.kumara@ubd.edu.bn (N.T.R.N.K.); cheeming.lim@ubd.edu.bn (C.M.L.); 2 Department of Optoelectronics and Materials Technology, National Taiwan Ocean University, Keelung 20224, Taiwan; suyang191@gmail.com; 3 Taiwan Instrument Research Institute, National Applied Research Laboratories, Hsinchu 300, Taiwan; hjhuang@narlabs.org.tw

**Keywords:** metal-insulator-metal, waveguide, rectangular cavity, silver baffles, finite element method

## Abstract

A plasmonic metal-insulator-metal waveguide filter consisting of one rectangular cavity and three silver baffles is numerically investigated using the finite element method and theoretically described by the cavity resonance mode theory. The proposed structure shows a simple shape with a small number of structural parameters that can function as a plasmonic sensor with a filter property, high sensitivity and figure of merit, and wide bandgap. Simulation results demonstrate that a cavity with three silver baffles could significantly affect the resonance condition and remarkably enhance the sensor performance compared to its counterpart without baffles. The calculated sensitivity (S) and figure of merit (FOM) in the first mode can reach 3300.00 nm/RIU and 170.00 RIU^−1^. Besides, S and FOM values can simultaneously get above 2000.00 nm/RIU and 110.00 RIU^−1^ in the first and second modes by varying a broad range of the structural parameters, which are not attainable in the reported literature. The proposed structure can realize multiple modes operating in a wide wavelength range, which may have potential applications in the on-chip plasmonic sensor, filter, and other optical integrated circuits.

## 1. Introduction

Surface plasmon polaritons (SPPs) can use as electromagnetic (EM) wave transportation and confine EM wave at the dielectric–metal interface because of their capacities of handling light in the nanoscale [1,2,3,4,5,6,7,8,9,10,11,12,13,14,15]. A standard dielectric (or insulator) waveguide cannot form the EM wave beyond conventional optics’ diffraction limit [16]. Waveguides compose of an insulator placed between two metals term as metal-insulator-metal (MIM) waveguides possessing transmitting SPPs modes that are strongly restricted in a dielectric region with an adequate propagation length of SPPs [17,18,19]. A traditional plasmonic MIM waveguide consists of the straight waveguides and the resonance cavities [20,21]. Taking advantage of MIM waveguides, various plasmonic devices can be designed and manufactured [22,23,24,25,26,27,28]. Recently, cavity resonance formed in MIM waveguides to obtain wavelength filtering and refractive index sensing functions has been investigated by theoretical estimations and experimental verifications [29,30,31,32,33,34,35,36,37]. Several resonance modes can be excited in the straight waveguide coupled with the resonance cavity under SPPs conditions, which are highly sensitive to the refractive index’s variation and the cavity’s shape [38,39,40,41,42,43].

The resonance cavity in a MIM plasmonic waveguide system with different structural aspects plays a crucial role to form the SPPs modes in the resonance cavity. Various resonance cavities in plasmonic MIM waveguide using single or multiple cavities have theoretically analyzed and numerically investigated [44,45,46,47,48,49,50,51,52]. Many efforts have focused on enlarging the cavity size to achieve high sensitivity as the effective cavity length has a linear relationship to the increased resonance wavelength [20,37,53]. Although the higher sensitivity can be obtained, the drawback of enlarging device size and manufacturing complexity are presented. 

Sensitivity and figure of merit are the two potential features for sensing performance. However, most efforts can only improve the above two factors in one spectrum channel, e.g., only one of the resonance modes has much higher sensitivity than the other modes [54,55,56,57,58,59]. Besides, most previous studies could not consider the influence of the cavity’s baffle effect in the resonance cavity and consider improving the two factors (i.e., S and FOM) in multiple channels (or multimode). The baffle’s influence could change the resonance condition that happened in the resonance cavity. The number of the geometrical parameters are suggested to be used as small as possible, since it is easier to optimize the parameters. The rectangular shape is the right choice because the straight edges compare to the figure with bent or complicated ones, e.g., ring shape, triangular, bowtie shape, and other combinations of different forms. 

In this paper, a plasmonic metal-insulator-metal (MIM) waveguide refractive index sensor consisting of a rectangular cavity, including three silver (Ag) baffles, is designed and analyzed. We calculate the transmittance spectrum and magnetic H_z_ field distributions using a two-dimensional (2-D) finite element method (FEM) and examine the resonance modes in the rectangular cavity by the cavity resonance mode theory. The obtained results are approximately in line with the analytical ones. The influence of the structural parameters on the transmittance properties, refractive index sensitivity, and figure of merit was also explored. The proposed structure shows a simple shape with a small number of structural parameters that can function as a plasmonic sensor with a filter feature. One can demonstrate that a cavity with three baffles could significantly affect the resonance condition and remarkably enhance the sensor performance compared to its counter without the baffles. The proposed structure can realize multiple modes operating in a wide wavelength range, which may have potential applications in the on-chip plasmonic sensor, filter, and other nanophotonic devices in highly integrated optical circuits.

## 2. Structure Design and Simulation Method

**Figure 1:**
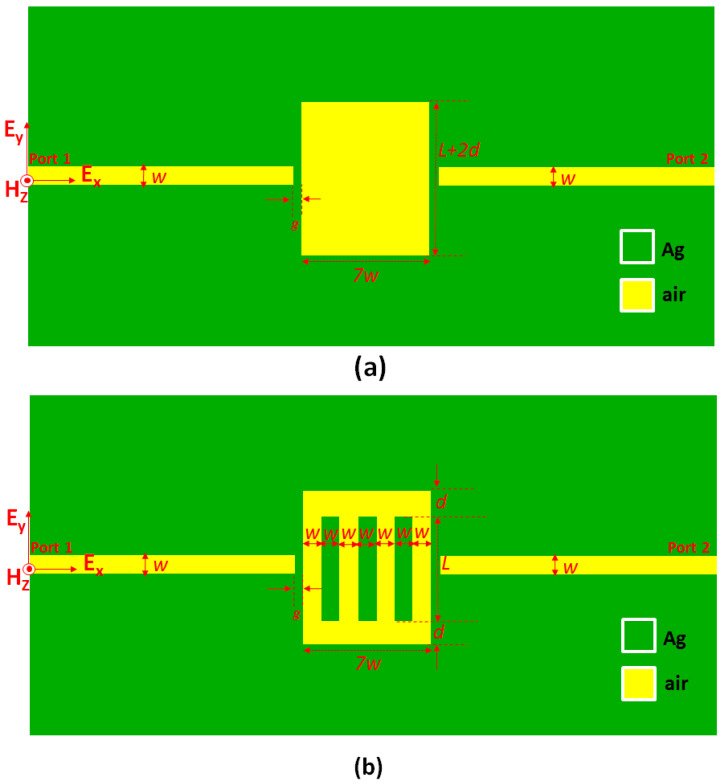
Schematic illustration of the proposed MIM waveguide containing the straight waveguides (width w), one centrally coupled rectangular cavity (width 7w and length *L* + 2*d*) (**a**) excluding and (**b**) including three Ag baffles in the rectangular cavity. The Ag buffers uniformly distribute in the rectangular cavity. The gap distance between the rectangular cavity and the straight waveguides is *g*.

Figure 1a,b depicts the schematic diagrams of the proposed plasmonic filters that consist of two straight waveguides and one central coupled rectangular cavity, excluding and including three silver (Ag) baffles with the same size. The Ag baffles (width w and length *L*) with adjacent gap distance w uniformly distribute in the rectangular cavity. The vertical coupling distance between the cavity edge and the Ag baffles is *d*. The straight waveguide width (w) is kept as 50 nm to ensure that the fundamental mode can transmit to the plasmonic MIM waveguide. Simulations were performed by a 2D FEM using the commercial software COMSOL Multiphysics (Version 5.2a, Stockholm, Sweden) [60] with perfectly matched layer absorbing boundary conditions to absorb the outgoing waves (to make sure that the light that is propagating out of the modeling domain does not get reflected). Note that a three-dimensional (3D) model is reduced to a 2D one since both models will obtain the similar results in simulations [49,61,62] and experiments [63,64], provided that the device height or thickness in the z-direction is much longer than in the x- and y- directions [52,65]. Therefore, we can ignore the effect of the device’s height on the obtained results by supposing that the device height is infinite [51]. Besides, the 2D approximation can speed up the simulation time and reduce the required computer resources, without sacrificing the calculation accuracy [66].

A TM-polarized incident EM wave with in-plane electric field component E*_x_* along the *x*-direction is coupled to the plasmonic MIM waveguide system. Therefore, only the fundamental transverse magnetic (TM_0_) mode can be propagated in the waveguide, supporting SPPs waves [67]. In the FEM simulations, the transmittance (*T*) of the MIM waveguide is *T* = (S_21_)^2^, where S_21_ is the transmission coefficient from the input end (i.e., port 1) to the straight’s output end waveguides (i.e., port 2).

The two plasmonic nanometals are gold (Au) and silver (Ag). We chose Ag as a metal material because of the cost-consuming method for the proposed structure’s future fabrication. The oxidation of Ag can be controlled using a thin layer of SiO_2_ be deposited on the top to prohibit the direct exposure of Ag with air. The simulation works can also be available for the other metals (e.g., Au, Pt, etc.). The data of frequency-dependent complex relative permittivity ε_m_ of Ag is referred to Drude model as shown in Equation (1), and the dielectric constant of Ag is appropriate to describe the optical properties of Ag in the large wavelength range, which is considered in this work [32,68,69].

(1) $$\varepsilon_{m}\left(\omega \right)=\varepsilon_{\infty }-\frac{\omega_{p}^{2}}{\omega^{2}+i\omega \gamma }\;,$$

where ω is the angular frequency of the incident EM wave. ε_∞_ is the dielectric constant at the infinite frequency (ε_∞_ = 3.7), ω_p_ is the electron collision frequency (ω_p_ = 1.38 × 10^16^ Hz = 9.10 eV), and γ is the bulk plasma frequency (γ = 2.37 × 10^13^ Hz = 18 meV). The resonance wavelength (λ_res_) based on the cavity resonance mode theory can be described by [70,71,72]:

(2) $$\lambda_{\mathrm{res}}=\frac{2\ell_{eff}Re(n_{\mathrm{eff}})}{m-\frac{\theta }{\pi }}\;\left(m=1,2,3\ldots \right),$$

where λ_res_ is the resonance wavelength at transmittance peak, *ℓ_eff_* is the effective cavity length, and *Re*(*n*_eff_) represents the real part of the effective refractive index. *m* is the mode number (positive number, i.e., *m* = 1, 2, 3,...), and *θ* is the phase. Based on the Equation (2), the λ_res_ can easily tune by varying the *ℓ_eff_* and *n*_eff_ of the rectangular cavity.

The sensitivity (S) can be calculated as S = Δλ/Δ*n* nanometer per refractive index (nm/RIU), where Δλ is the shift of the resonance wavelength of transmittance, λ_res_ is the resonance wavelength at transmittance peak, and Δ*n* is the refractive index difference. The figure of merit (FOM) is defined as S/FWHM, where FWHM is the full width at half-maximum of the transmittance spectrum. Besides, the quality factor can be defined as Q = λ_res_/FWHM.

Thanks to the fast progress in nanophotonic, the fabrication of the proposed structure is achievable with current technologies [73,74,75,76,77,78,79,80,81,82,83,84,85,86,87,88], allowing the cost-effective fabrication over a large area. A similar structure of a MIM waveguide with a central-coupled rectangular cavity has previously been fabricated using physical vapor deposition and a focused ion beam for etching the rectangular cavity [79]. Another technology has also been proposed in [80], in which the straight waveguides and the rectangular cavity are formed by electron beam lithography. Moreover, wet chemical etching or vapor deposition techniques can provide a simple method for producing metal baffles [80,81]. However, the aim of this article is not to focus on fabrication methods. As an alternative, several potential papers that investigate in-depth coverage of this vital subject are suggested [76,77].

## 3. Results and Discussion

**Figure 2:**
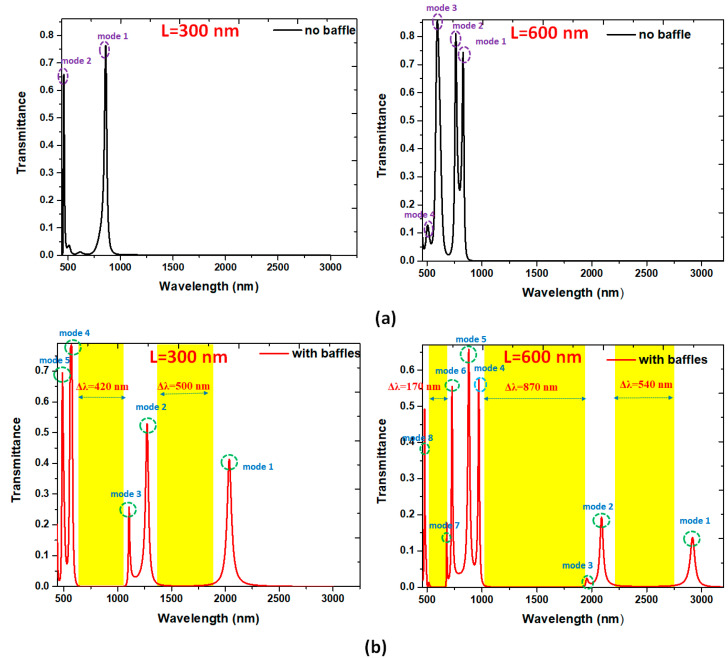
Transmittance spectra of the proposed plasmonic filter (**a**) without (black color) and (**b**) with (red color) Ag baffles in the rectangular cavity for *L* = 300 nm and *L* = 600 nm, respectively. The structural parameters *w*, *g*, and *d* are 50, 10, and 30 nm, respectively.

The proposed MIM waveguide plasmonic filter can serve as band-pass and band-stop filters, which permits and prohibits transmitting specific wavelengths of EM wave. However, narrow FWHM in pass-band and broad bandgap in stop-band is desirable in this plasmonic filter. We compared the proposed structures’ transmittance spectra without and with Ag baffles, as shown in Figure 2a,b, respectively. We use air (*n* = 1) as an insulator in the straight waveguides and rectangular cavity for simplicity. Two lengths of Ag baffles, i.e., *L* = 300 and *L* = 600 nm, are chosen for comparison. The other structural parameters, *w*, *g*, and *d*, are 50, 10, and 30 nm, respectively. An evident difference after the different lengths of Ag baffles are introduced in the MIM waveguide system, and the resonance condition difference can interpret this in the rectangular cavity. As seen in Figure 2a,b, only the Ag baffles cases reveal an apparent band-pass and band-stop profiles. More resonance modes could happen when the bigger cavity (i.e., longer *L*) is used. The Ag baffle case’s transmittance spectrum shows a prominent filtering characteristic with a narrow FWHM at the corresponding resonance modes in the rectangular cavity. In Figure 2a, two clear transmittance peaks were found at λ_res_ = 859 and 460 nm (marked by mode 1 and mode 2) concerning the case without Ag baffles for *L* = 300 nm and four ones were found at λ_res_ = 829, 762, 594, and 506 nm (marked by mode 1 to mode 4) concerning the case without Ag baffles for *L* = 600 nm, correspondingly. In Figure 2b, five clear transmittance peaks appeared at λ_res_ = 2036, 1273, 1106, 570, and 487 nm (marked by mode 1 to mode 5) and eight ones appeared at λ_res_ = 2916, 2088, 1955, 972, 880, 728, 680, and 478 nm (marked by mode 1 to mode 8) concerning the case with Ag baffles for *L* = 600 nm, correspondingly. These results show a noticeable increase in the λ_res_ when the Ag baffles are set in the rectangular cavity and the length of Ag baffles is increased. The case with Ag baffles (Figure 2b) shows a better performance compared to the case without Ag baffles (Figure 2a). The working wavelengths in the case without Ag baffles are in visible range, whereas in the case with Ag baffles, they can spread both in visible and near-infrared spectra. It is worth noting that the very sharp transmittance peaks can be seen in Figure 2a,b, indicating the proposed structure with the feature of high quality factor (Q factor). The calculated Q factors are 145.80, 139.20, 130.33, 97.2, 68.2, 176.00, 182.00, and 159.33 of mode 1 to mode 8, respectively, concerning the case with Ag baffles for *L* = 600 nm.

These transmittance peaks do not reach unity because of the metal’s internal loss in the proposal MIM waveguide system. Note that the SPP modes’ loss in larger wavelengths is more significant than those in smaller ones. This phenomenon is due to the SPP modes in larger wavelengths having more optical path and substantial power leaks from the MIM waveguides than the smaller ones, i.e., short wavelengths could decrease optical waveguide power consumption [82]. At λ_res_, the SPPs mode in the rectangular cavity can be excited, and the EM wave can be transmitted. At the bandgap region (transmittance trough), the incident EM wave can be ultimately reflected.

In Figure 2b, there are two bandgap regions for *L* = 300 nm with bandgap width of Δλ = 420 and 500 nm, and three ones with bandgap width of Δλ =170, 870, and 540 nm for *L* = 600 nm, which does not appear in the case without baffles (i.e., no bandgap region between two transmittance peaks). Note that the bandgap width is significantly higher than those of the reported works [83,84]. In the proposed MIM waveguide, the transmittance raised maximum as the SPP modes fit the resonance condition in the rectangular cavity.

Based on Equation (2), λ_res_ is closely related to the *n*_eff_ and *ℓ_eff_* of the resonance cavity. The existence of Ag baffles may lead to the larger *ℓ_eff_* and *n*_eff_ for increasing λ_res_ (i.e., the redshift). It is worth noting that more transmittance peaks (or SPPs modes) and transmittance troughs (or bandgap regions) are preferred in the single plasmonic MIM waveguide system to fit the design of a miniaturized integrated optical circuit. The case with Ag baffles has more number of SPPs modes, which is superior to that of a small number of SPPs modes of the case without Ag buffers. The proposed structure with Ag baffles can be used as a multichannel on-chip plasmonic sensor with a filter function, and it also fits the requirement of the integrated optical circuits.

**Figure 3:**
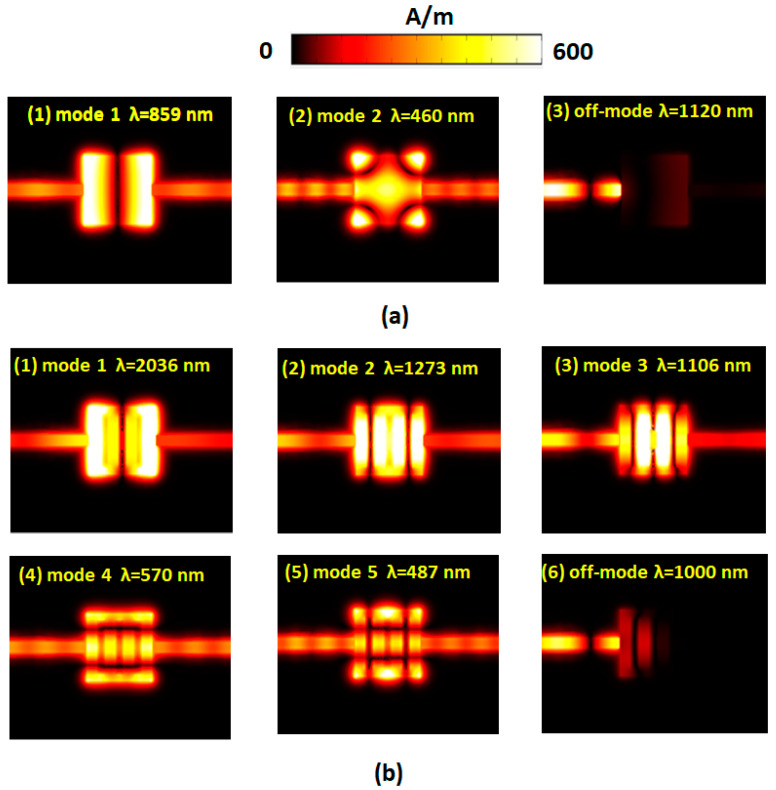
Truncate views of magnetic field intensity (|***H***|) for the cases (**a**) without Ag baffles at λ_res_ = 859 and 460 nm and (**b**) with Ag baffles at λ_res_ = 2036, 1273, 1106, 570, and 487 nm, concerning *L* = 300 nm, respectively. The off-resonance modes for the case without Ag baffles (at λ = 1120 nm) and the case with Ag baffles (at λ = 1000 nm) are also illustrated for comparison.

**Figure 4:**
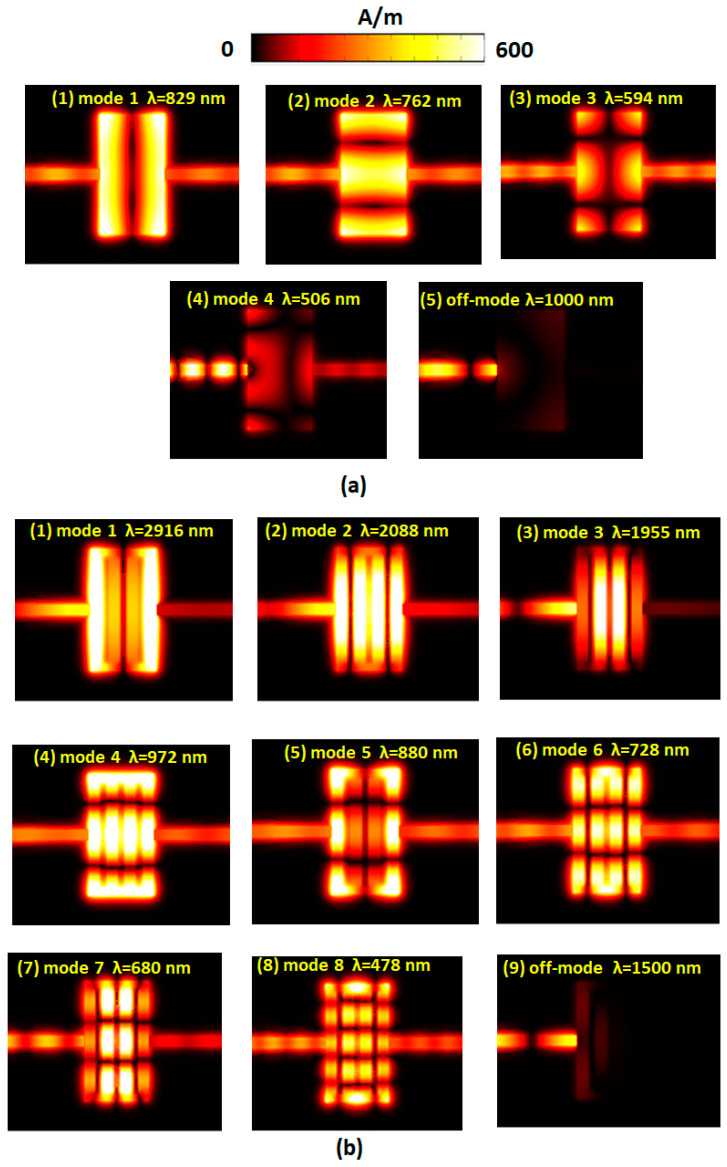
Truncate views of magnetic field intensity (|***H***|) for the cases (**a**) without Ag baffles at λ_res_ = 829, 762, 594, and 506 nm and (**b**) with Ag baffles at λ_res_ = 2916, 2088, 1955, 972, 880, 728, 680, and 478 nm concerning *L* = 600 nm, respectively. The off-resonance modes for the case without Ag baffles (at λ = 1000 nm) and the case with Ag baffles (at λ = 1500 nm) are also illustrated for comparison.

The SSPs modes found in Figure 2 are strongly dependent on the hybrid plasmonic effects of the gaps, cavity, and metal surfaces in the proposed MIM waveguide system [85,86,87,88,89]. The transmittance spectrum reveals a noticeable filtering feature at the corresponding resonance modes and bandgap regions. To interpreted the nature of Figure 2, we illustrated the magnetic field intensities (|***H***|) for the cases without and with the Ag baffles in the rectangular cavity at corresponding on-resonance modes (i.e., *λ*_res_) and off-resonance modes concerning *L* = 300 nm (Figure 3a,b) and *L* = 600 nm (Figure 4a,b), respectively. The rectangular cavity can function as a Fabry–Pérot cavity, indicating that the EM waves can be transmitted through the rectangular cavity only when resonating with each other. As can be seen in band-pass cases in Figure 3 and Figure 4 that almost the SPPs wave is coupled to the rectangular cavity well at *λ*_res_, and the |***H***| profiles show an entirely different patterns between the two cases. The mode patterns of |***H***| profiles are closely related to the size of incident wavelengths, i.e., shorter wavelength possesses more field lobes, whereas larger wavelength has fewer field lobes.

In the case with Ag baffles, the positive–negative charge pairs (or dipoles) distribute on the edge of the rectangular cavity surface and Ag baffles’ surface, resulting in the remarkable gap and cavity resonance compared to the case without Ag baffles. Besides, the case with Ag baffles contributes more dipoles, which will be beneficial for the coupling effects between nanometals and incident EM waves. Longer *L* could lead to more charge pairs on Ag baffles’ surface and the cavity edges. This characteristic can verify the |***H***| profiles in the case with Ag baffles (Figure 3b and Figure 4b), which show higher light enhancement and confinement between the metals in comparison to its counterpart without Ag baffles (Figure 3a and Figure 4a). In the case with Ag baffles, the EM waves can remarkably enlarge by the excitation of SPPs wave and the EM waves’ discontinuity across the straight waveguide interface, the rectangular cavity, and the Ag baffles. The resonance wavelength can be excited to the resonance mode, and the incident EM wave can be conveyed from the input end to the output end. As can be observed, the off-resonance mode in Figure 3 and Figure 4, the incident mode can be ultimately reflected at the left part of the straight waveguide and cavity (see Figure 3(a3,b6) and Figure 4(a5,b9)).

**Figure 5:**
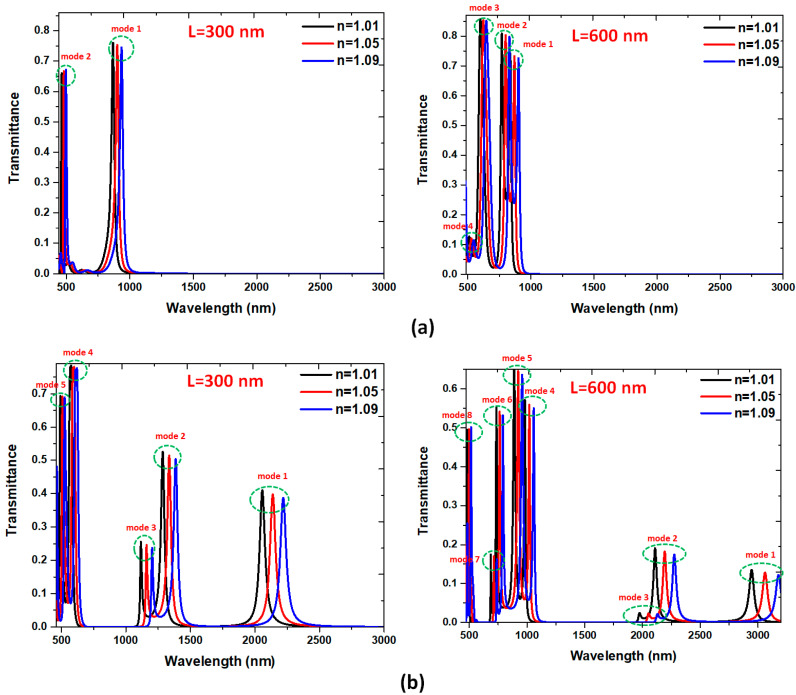
Transmittance spectra of the proposed plasmonic sensors (**a**) without and (**b**) with Ag baffles concerning *L* = 300 nm and *L* = 600 nm. The refractive index (n) is varied from 1.01, 1.05 to 1.09 at the interval of 0.04, respectively, while the other structural parameters w, *g*, and *d* are 50, 10, and 30 nm, respectively.

The proposed plasmonic filter can also apply as a refractive index sensor when the surrounding medium’s refractive index is changed. Figure 5a,b shows the proposed structure’s transmittance spectra without/with Ag baffles concerning *L* = 300 nm and *L* = 600 nm. To test the device’s sensitivity performance, we chose the refractive index values in the range of 1.01–1.09, and the other range of refractive index values has the same trend of transmittance spectrum based on our simulations [20]. The refractive index (*n*) is varied from 1.01, 1.05 to 1.09 at the interval of 0.04, respectively, while the other structural parameters w, *g*, and *d* are 50, 10, and 30 nm, respectively. As observed in Figure 5a,b, the transmission peaks show a redshift and a linear relationship with increasing *n*, which is in line with Equation (2). The redshift increase is due to the enhancing EM wave in a rectangular cavity, mostly interacting with the refractive index change. Since there is hybridization of the waveguide mode and the gap and cavity plasmon resonance modes in the rectangular cavity, a small refractive index change (Δ*n*) results in a significant wavelength shift.

**Figure 6:**
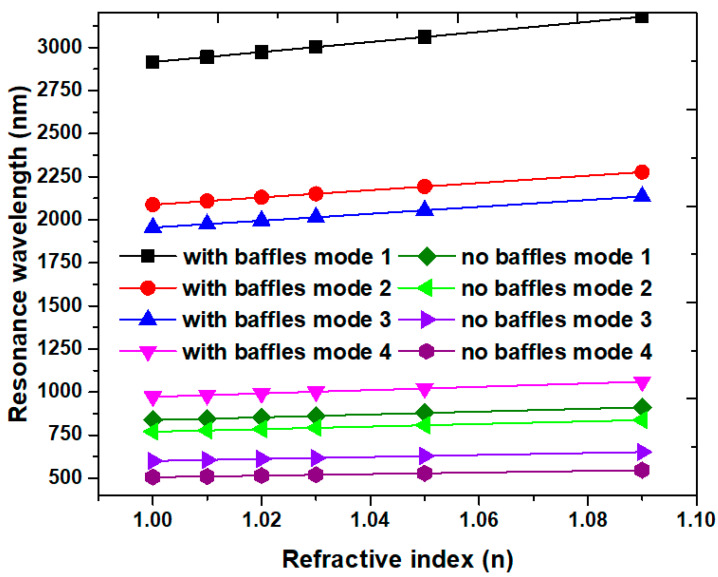
Illustration of the resonance wavelength (λ_res_) versus the refractive index (n) of the cases without and with Ag baffles concerning *L* = 600 nm in mode 1 to mode 4, respectively. The other structural parameters w, *g*, and *d* are 50, 10, and 30 nm.

A suitable refractive index sensor simultaneously possesses the high sensitivity (*S*) and figure of merit (FOM) [90,91,92,93,94,95,96]. Figure 6 illustrates the resonance wavelength (λ_res_) versus the refractive index (*n*) of the cases without and with Ag baffles concerning *L* = 600 nm in mode 1 to mode 4. The other structural parameters w, *g,* and *d* are 50, 10, and 30 nm. It found that a redshift of λ_res_ increases *n*, with a more massive shift in mode 1 and mode 2 than in other modes. Results show that the *n* can be estimated easily by certain λ_res_ according to Equation (2), demonstrating that the proposed structure’s features can function as a refractive index sensor. The S and FOM of the case without Ag baffles are 825.00 nm/RIU and 165.00 RIU^−1^ for mode 1, 750.00 nm/RIU and 150.00 RIU^−1^ for mode 2, 575.00 nm/RIU and 57.50 RIU^−1^ for mode 3, and 450.00 nm/RIU and 56.25 RIU^−1^ for mode 4, correspondingly. The higher average values of S and FOM can be achieved in the case with Ag baffles, i.e., 2900.00 nm/RIU and 145.00 RIU^−1^ for mode 1, 2100.00 nm/RIU and 140.00 RIU^−1^ for mode 2, 2000.00 nm/RIU and 200.00 RIU^−1^ for mode 3, 1000.00 nm/RIU and 200.00 RIU^−1^ for mode 4, 900.00 nm/RIU and 180.00 RIU^−1^ for mode 5, 700.00 nm/RIU and 175.00 RIU^−1^ for mode 6, 600.00 nm/RIU and 200.00 RIU^−1^ for mode 7, and 600.00 nm/RIU and 300.00 RIU^−1^ for mode 8, correspondingly. Compared to the case without Ag baffles, the case’s performance with Ag baffles in the rectangular cavity generates an enhancement of filter’s sensitivity by 251.50% for mode 1, 180% for mode 2, 247.83% for mode 3, and 122.22% for mode 4. These values are remarkable and highly satisfy the request of plasmonic refractive index sensor. It is worth noting that it can simultaneously achieve higher sensitivity and figure of merit, which are larger than 2000.00 nm/RIU and 140.00 RIU^−1^ in mode 1 to mode 3 in our design. The proposed structure is appropriate for working as a gas sensor or in a liquid environment. The reason is that the gas sensors usually require higher sensitivity since the refractive index changes are small, while lower sensitivity might be tolerable in water. 

**Figure 7:**
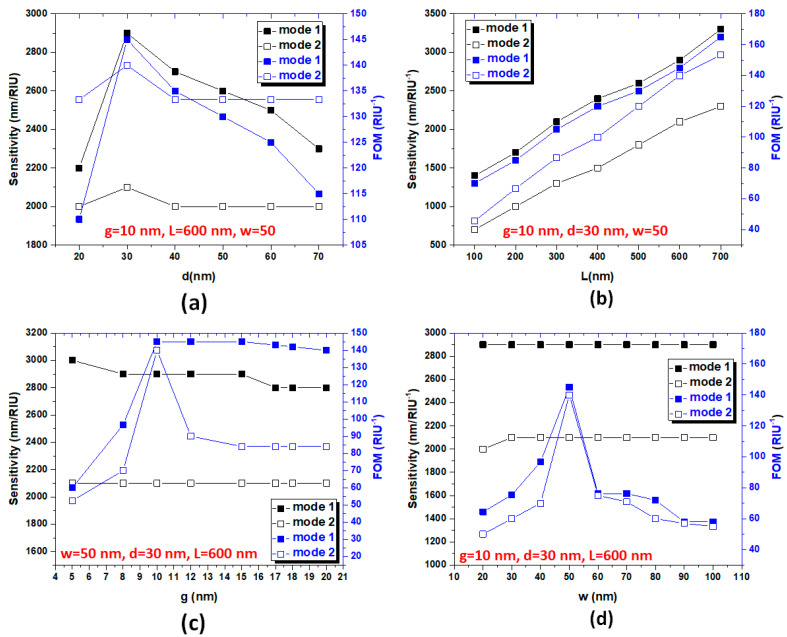
Sensitivities and figure of merit (FOM) of the proposed plasmonic sensor with Ag baffles in mode 1 and mode 2 for varying (**a**) vertical coupling distance (*d*) from 10 to 70 nm in the step of 10 nm, (**b**) varying Ag buffalo length (*L*) from 300 to 700 nm in the step of 100 nm, (**c**) varying coupling distance (*g*) in the range of 5–20 nm, and (**d**) varying the straight waveguide width (w) from 20 to 100 nm in the step of 10 nm, correspondingly. The other structural parameters are indicated in the inset of the figures.

Subsequently, we discuss the influence of structural parameters on sensitivities and figure of merit of the proposed structure. For simplifying the optimization of the structural parameters, the number of structural parameters is used as small as possible. Fortunately, only four structural parameters (i.e., w, *g*, *d*, and *L*) are considered in our design. In the previous simulations, we fixed w = 50 nm for the fundamental mode to excite the straight waveguide and *g* = 10 nm for efficient coupling between the straight waveguide and the cavity [20,32]. Next, the four structural parameters, *d, L*, w, and g, will be inspected in detail. The air region’s resonance condition in the rectangular cavity can be changed by varying the value of *d* and *L* since the air region between Ag baffles, and the cavity edges has been changed, resulting in a variation of *n*_eff_ and *ℓ_eff_* in the resonance cavity. Similarity, the straight waveguide width (w) and horizontal coupling distance (*g*) could affect the propagating mode in the straight waveguide and resonance condition in the rectangular cavity. Figure 7a,b shows the sensitivities and figure of merit of the proposed plasmonic sensor with Ag baffles in mode 1 and mode 2 for varying coupling distance (*d*) from 10 to 70 nm in the step of 10 nm and for varying Ag baffle’s length (*L*) from 300 to 700 nm in the step of 100 nm, respectively. The other structural parameters are denoted in the inset of the figures. In Figure 7a, the variation of *d* in the range of 20–70 nm demonstrates that the proposed structure’s sensitivity and FOM simultaneously achieve above 2000.00 nm/RIU and 110.00 RIU^−1^ in modes 1 and 2, revealing the robustness of manufacturing. These results with high sensitivity and figure of merit in mode 1 and mode 2 cannot be simultaneously obtained in the reported literature (e.g., [97,98,99,100,101,102]). The optimal value is *d* = 30 nm, which can support the plasmon resonance mode to enhance the proposed structure’s sensitivity and FOM. As can be seen in Figure 7b, the Ag baffle’s length (*L*) can significantly influence the sensitivity and figure of merit with *L*’s increase when *L* is in the range of 100–700 nm. The highest sensitivity can reach *S* = 3300.00 nm/RIU along with a high FOM of 170.00 RIU^−1^ and ultrawide bandgap (results not shown here). According to the simulations and analysis above, the proposed plasmonic filter’s function can easily tune by changing the *d* and *L* set in the rectangular cavity. Therefore, one can vary *d* and *L* in the rectangular cavity to design the band-pass and band-stop plasmonic filter with the desired working wavelength.

**Figure 8:**
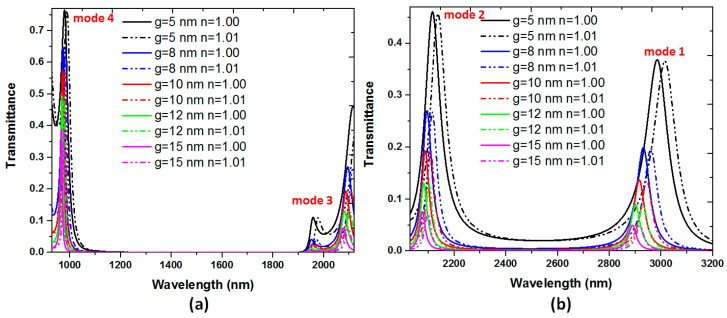
Transmittance spectrum of the proposed plasmonic filter of (**a**) mode 3 and mode 4 and (**b**) mode 1 and mode 2 versus different coupling distances (*g*) and different refractive indices (*n*). The *g* values are varied from 5, 8, 10, 12 to 15 nm and *n* values change from 1.00 to 1.01.

Figure 7c,d shows the sensitivities and figure of merit of the proposed plasmonic sensor with Ag baffles in mode 1 and mode 2 for varying coupling distance (*g*) in the range of 5–20 nm and for varying the straight waveguide width (w) from 20 to 100 nm in the step of 10 nm, respectively. In Figure 8a, b the structural parameters *g* and w can also affect the proposed structure’s SPPs mode as well as resonance condition. As observed, the sensitivity shows the same trend and simultaneously reaches above 2000.00 nm/RIU in mode 1 and mode 2 in a wide range of *g* and w, while the FOM reveals the different values due to the different FWHM. Based on our simulations, the higher transmission can generate a larger extinction ratio and a smaller FWHM, contributing to a larger FOM. In Figure 7c,d, the optimal values are *g* = 10 nm and w = 50 nm, respectively. These features found in Figure 7a–d can explain by the increase in *ℓ_eff_* and *n_eff_* in the rectangular cavity, as indicated in Equation (2). It is worth noting that a problem on how well can these FOM and S be reproduced by an experimental structure and given a fabrication tolerance. The suggested structural parameters of the proposed plasmonic sensor are in the range of 20 nm < *d* <70 nm, 300 nm < *L* < 700 nm, 5 nm < *g* < 15 nm, and 40 nm < w <70 nm, respectively. The high sensitivity and figure of merit can also be achieved in a broad-spectrum range for promising applications in nanophotonics. With the recent advances in plasmonic sensors, there is an anticipation of enhancing sensitivity and reducing the detection limit to overcome the limitations in ultrasensitive sensing of biological and chemical analytes, especially at single molecule levels [103,104,105].

Finally, we investigate in more detail the influence of coupling distance *g* on the transmittance, since the structural parameter *g* of 10 nm would be rather difficult to control experimentally [78]. Figure 8 depicts the transmittance spectrum of the proposed plasmonic filter of mode 3 and mode 4 (Figure 8a) and mode 1 and mode 2 (Figure 8b) versus different coupling distances (*g*) and different refractive indices (*n*). The *g* values are varied from 5, 8, 10, 12 to 15 nm, and *n* values are changed from 1.00 to 1.01. We focus on the transmittance peaks and bandgap width in modes 1 to mode 3, while the other structural parameters are kept as w = 50, *d* = 30 nm, *L* = 600 nm, respectively. As observed, the transmittance peaks decrease with the increase of *g* because of the less coupling and more propagation loss between the straight waveguides and the rectangular cavity. Besides, the resonance wavelengths reveal slight blueshift to shorter wavelengths for a larger *g*, which well agrees with the reported literature [106]. The transmittance shift is about 20, 29, 29, 29, and 29 nm in mode 1 and 21, 21, 21, 21, and 21 nm in mode 2, when *g* varies from 5, 8, 10, 12 to 15 nm, correspondingly. In these cases, the sensitivity in mode 1 and mode 2 can simultaneously exceed 2000.00 nm/RIU when *g* in the range of 5–15 nm, indicating that the proposed structure possesses a high fabrication tolerance. It is evident that the parameter *g* is not only affecting the transmittance spectrum but also influences the transmission bands’ line shape. As seen in Figure 8b, the bandgap width between mode 1 and mode 2 is increased as the *g* value increases, while the bandgap width between mode 3 and mode 4 remains unchanged (see Figure 8a). It is also important to note that, there is no bandgap (or transmittance through) between mode 1 and mode 2 when *g* = 5 nm, since a narrow coupling distance leads to more coupling effect between the straight waveguide and the rectangular cavity.

## 4. Conclusions

In conclusion, we have proposed a simple designed plasmonic MIM filter with a high sensitivity and a high figure of merit as well as a wide bandgap. The proposed structure consists of two straight waveguides, three Ag baffles, and one rectangular cavity. The sensitivity and figure of merit of the structure can be improved by varying two structural parameters, i.e., the length of Ag baffles (*L*) and the coupling distance between the baffles and cavity (*d*). Results show the obvious on-resonance and off-resonance modes and narrow FWHM in the proposed structures due to the hybrid plasmonic coupling effect of the metal’s surfaces, gaps, and cavities. The proposed design has a simple shape with a small number of structural parameters, making it easy for integration and optimization. The highest sensitivity of 3300.00 nm/RIU and figure of merit of 170.00 RIU^−1^ can be achieved. The variation of *d* in the range of 20–70 nm demonstrates that the proposed structure’s sensitivity and figure of merit can simultaneously achieve above 2000.00 nm/RIU and 110.00 RIU^−1^ in the first and second modes, which cannot obtain from the reported literature. FEM simulations investigated the simulation results, and it believed that the proposed structure can support full application in the on-chip optical sensing and filtering areas. Besides, these simulation results obtained from FEM are in good agreement with the cavity resonance mode theory.
